# Development of An Artificial Male Germ Cell Niche Using Electrospun Poly Vinyl Alcohol/Human Serum Albumin/Gelatin Fibers 

**DOI:** 10.22074/cellj.2019.6120

**Published:** 2019-06-15

**Authors:** Zahra Borzouie, Majid Naghibzadeh, Ali Reza Talebi, Fatemeh Pourrajab, Ali Jebali, Habib Nikukar, Hosein Molla Hoseini, Arezoo Khoradmehr, Fatemeh Sadeghian-Nodoushan, Behrouz Aflatoonian, Seyedhossein Hekmatimoghaddam, M.D. 1

**Affiliations:** 1 Stem Cell Biology Research Center, Yazd Reproductive Sciences Institute, Shahid Sadoughi University of Medical Sciences, Yazd, Iran; 2 Research and Clinical Center for Infertility, Yazd Reproductive Sciences Institute, Shahid Sadoughi University of Medical Sciences, Yazd, Iran; 3 Department of Biochemistry and Molecular Biology, School of Medicine, Shahid Sadoughi University of Medical Sciences, Yazd, Iran; 4 Department of Advanced Medical Sciences and Technologies, School of Paramedicine, Shahid Sadoughi University of Medical Sciences, Yazd, Iran; 5 Textile Engineering Faculty, Isfahan University of Technology, Isfahan, Iran; 6 Medical Nanotechnology and Tissue Engineering Research Center, Yazd Reproductive Sciences Institute, Shahid Sadoughi University of Medical Sciences, Yazd, Iran; 7 Department of Reproductive Biology, School of Medicine, Shahid Sadoughi University of Medical Sciences, Yazd, Iran

**Keywords:** Azoospermia, Human Serum Albumin, Scaffold, Testis, Tissue Engineering

## Abstract

**Objective:**

Recent achievements in stem cell biotechnology, nanotechnology and tissue engineering have led to development of novel approaches in regenerative medicine. Azoospermia is one of the challenging disorders of the reproductive system. Several efforts were made for isolation and culture of testis-derived stem cells to treat male infertility. However, tissue engineering is the best approach to mimic the three dimensional microenvironment of the testis in vitro. We investigated whether human testis-derived cells (hTCs) obtained by testicular sperm extraction (TESE) can be cultured on a homemade scaffold composed of electrospun nanofibers of homogeneous poly (vinyl alcohol)/human serum albumin/gelatin (PVA/HSA/gelatin).

**Materials and Methods:**

In this experimental lab study, human TCs underwent two steps of enzymatic cell isolation and five culture passages. Nanofibrous scaffolds were characterized by scanning electron microscopy (SEM) and Fourier- transform infrared spectroscopy (FTIR). Attachment of cells onto the scaffold was shown by hematoxylin and eosin (H&E) staining and SEM. Cell viability study using MTT [3-(4, 5-dimethyl-2-thiazolyl) -2, 5-diphenyl -2H- tetrazolium bromide] assay was performed on days 7 and 14.

**Results:**

Visualization by H&E staining and SEM indicated that hTCs were seeded on the scaffold. MTT test showed that the PVA/HSA/gelatin scaffold is not toxic for hTCs.

**Conclusion:**

It seems that this PVA/HSA/gelatin scaffold is supportive for growth of hTCs.

## Introduction

Almost 7% of all men, including those who lack spermproduction, suffer from infertility (1). Stem cells, with theirgreat and unique capacity to form other cell types, have raisedhuge hopes for scientists and clinicians as well as patientswith male infertility. After the derivation of mouse embryonicstem cells (2), different studies used mouse primordial germcells (PGCs) to investigate the biology of germ cells and theirprogenitors (3, 4). Later, in 1998, the first pluripotent stemcells were generated from pre-implantation human embryosin blastocyst stage (5), and also human PGCs (6) which werenamed human embryonic stem cells (hESCs) and humanembryonic germ cells (hEGCs), respectively. Since 2003, several studies have shown the potential of the ESCs to formmale and female germ cells (7-10). However, no gametehas been produced so far. Some investigations made effortsto reprogram unipotent spermatogonial stem cells (SSCs) to derive pluripotent germ-line stem cells (GSCs) in vitro in mice (11), rats (12) and humans (13). Nonetheless, laterreports indicated that human testis-derived cells (hTCs) are not pluripotent and possess characteristics similar to those of mesenchymal stromal cells (14). The latest studies includingIrie and Surani’s investigations (15) revealed that germ celldevelopment in humans differs from that in mice especiallyin terms of gene expression profile, which might be thereason for variations in results. Despite improvements inthe field, there are still challenges for translation of stem cell biotechnology to bedside practice (i.e. has not yet been used in male reproductive/regenerative medicine). 

In a recent study, mouse fertile sperm production from GSCs was done using organ culture (16). Besides developmental differences between mouse and human germ cells, there are more restrictive ethical issues regarding human organ culture compared to mouse organ culture. Therefore, tissue engineering methods are highly required for regeneration of some tissues and organs. These methods prepare bio-scaffolds to promote the development of new tissues such as cartilage or bone. In comparison with other instances where tissue engineering produced artificial tissues, researchers in the field of human male infertility could not obtain adequate mature cells. In regenerative medicine, utilization of pluripotent or multipotent stem cells has higher chance of success compared to unipotent cells like human SSCs (17). 

We previously showed the mulipotency of hTCs obtained from TESE samples (14). The aim of this study was to make a homemade scaffold composed of electrospun fibers of homogeneous solution of poly (vinyl alcohol)/ human serum albumin/gelatin (PVA/HSA/gelatin) as a niche for hTCs. Development of an artificial organ culture for production of male germ cells from hESC-derived GSCs could be the ultimate goal in this field.

## Materials and Methods

### Fabrication of the scaffold

In this experimental lab study, initially 450 mg of PVA powder (Merck, Germany, MW 72,000) was dissolved in deionized water (to reach a final concentration of 7% w/v) in a final volume of 6 mL which was kept at 80°C for 5 hours in a sterile beaker to make a clear solution. Next, 0.3 g gelatin powder (Merck, Germany) was added and the mixture was mixed by a magnetic stirrer at room temperature (RT). Then, 2 mL of a 20 g/dL solution of HSA (CSL Behring AG, Switzerland) was added to the mixture and mixed for 60 minutes on a magnetic stirrer. The resulting solution was homogenous and milky white.

The prepared homogeneous PVA/HSA/gelatin solution was electrospun into fibers using Electroris (FNM Ltd., Iran). The instrument consisted of a high voltage power supply, a conductive collector, a reservoir of polymer solution, and a nozzle with adjustable distance to collector.

To produce electrospun fibers, the polymer solution wasdrawn into a 5 mL syringe with a metallic needle of 0.4 mminternal diameter. The syringe was kept horizontally on thesyringe stand with the metal needle tip being connected tothe positive electrode of the high voltage power supply. Thevoltage was set at 16 KV, and the distance from collectorwas 10 cm. The experiment was done at RT (25°C) (18). The fibers were collected after 3 hours on circular glasscoverslips. The obtained PVA/albumin/gelatin fibrous scaffold was further cross-linked in glutaraldehyde vapor atRT for 1 day, then immersed in deionized water to removethe glutaraldehyde. The cross-linked scaffolds were dried and prepared for testicular cells culture (19). 

### Chemical analysis of scaffolds

Fourier-transform infrared (FTIR) spectroscopy conducted over a range of 4000-500 cm^-1^ was used for analysis of the PVA/HSA/gelatin fibrous scaffolds. The Nicolet spectrometer system (BOMEM FTIR MB-series, MB-100, Hartmann & Braun, Canada) provided FTIR spectra using a DTGS KBr detector. For this, about 1 mg of dried scaffold was mixed with 100-120 mg of KBr to make compressed pellets.

### Determination of scaffolds’ hydrophilicity, morphology, fiber diameter and pore size 

Before and after exposure to glutaraldehyde, water contact angles of electrospun scaffold were measured by a video-based optical system (model MV500 digital microscope, EasyTear, Italy). The images of water drops on the PVA/HSA/gelatin scaffold surface from three different angles were captured by the camera and analyzed by Digimizer image analysis software (MedCalc Software bvba, Belgium) to assess hydrophilicity. The volume of each water droplet was 5 µL, and measurements were done 10 seconds after contact. 

To evaluate the attachment of hTCs onto fibers, we performed hematoxylin (Merck, Germany) and eosin (Merck, Germany) (H<E) staining on scaffolds, on glass slides on days 7 and 14. 

The morphology of the scaffold was also characterized by scanning electron microscopy (SEM, model Phenom ProX, Phenom-World, The Netherlands) with an accelerating voltage of 15 kV after coating with gold. The average diameter of fibers and pore sizes were randomly determined by image analysis software (ImageJ, National Institute of Health, USA) to analyze 100 different fibers in each SEM image. 

### Sample collection and patients’ information

TESE samples were collected after obtaining signed informed consent from two patients with non-obstructive azoospermia attending a clinic for assisted reproduction. This study was approved by Ethics Committee of Shahid Sadoughi University of Medical Sciences, Yazd, Iran with reference No. IR.SSU.REC.1394.226. These two patients were chosen because their biopsies proved to contain germ cells. The fresh samples (about 40 mg each) were labeled with codes to maintain patient anonymity, placed in 2 mL of Dulbecco’s Modified Eagle Medium containing 5% fetal bovine serum (DMEM/5% FBS) (Invitrogen, UK), and transferred to the laboratory within 15 minutes (14).

### Preparation of human testis-derived cells from TESE samples

Approximately 30-40 mg pieces of the TESE samples were washed in DMEM medium and mechanically and enzymatically [collagenase type IV (Invitrogen, UK)] digested overnight using a previously reported protocol (14). The cells were subsequently recovered by aspiration, washed with DMEM and centrifuged for 3 minutes at 200 g. The supernatant was discarded, and the pellet was used for hTCs culture.

### Culture of human testis-derived cells 

The initial culture protocol was previously described by Sadeghian-Nodoushan et al. (14). Single-cell suspensions were placed in dishes with 45 mL of DMEM supplemented with 5 mL FBS, 100 ng/mL glial cell-derived neurotrophic factor (GDNF, R&D Systems, USA), and 20 ng/mL epidermal growth factor (EGF, R&D Systems, USA). Mostof the testicular cells were attached to the dish floor the dayafter initial extraction, and about 50% of the culture medium was exchanged every other day. Enzymatic treatment usingtrypsin (Sigma, Germany), and EDTA (Invitrogen, UK) wasperformed at 37°C for 3 minutes for passaging the hTCs. All cell culture experiments were performed at least in triplicate. 

### Transfer of human testis-derived cells on scaffold

The scaffolds were sterilized by one-hour UV irradiation. After five passages of hTCs in dishes, the cells were disaggregated using trypsin/EDTA, enumerated using a hemacytometer slide, plated on the scaffold at a concentrationof 5×10^3^ cells/coverslip placed in sterile dishes, and maintained at 34°C with 5% CO_2_. The cell-coated scaffolds were checked for cell proliferation/viability by the MTT [3(4, 5-dimethyl-2-thiazolyl) -2, 5-diphenyl -2H- tetrazolium bromide] test on days 7 and 14.

### Cell viability and proliferation assay (MTT assay)

To evaluate the viability and proliferation rate of thehTCs on the scaffold, we used the MTT test as a standard colorimetric assay which assesses cell viability based on themitochondrial dehydrogenase activity. Briefly, on days 7 and14, following cell incubation with and without scaffolds, 40µL of MTT solution (5 mg/mL in RPMI) was added to eachcentral well (containing coverslips covered by the scaffoldcontaining mixed testicular cells); then, the supernatant wasremoved, and 400 µL of 0.1 M HCl (prepared in isopropylalcohol) was added to dissolve formazan crystals. The opticaldensities (OD) at 570 nm (with background subtraction at630 nm) were evaluated using an ELISA (enzyme-linkedimmunosorbent assay) reader (Tajhizat Sanjesh, Iran). Percentage of viability and proliferation was determined by the following formula:

Percentage of viability=Optical density (OD) of the test sampleOD of the control samplex100

Any proliferation or decrease in the number of cells inscaffolds would so have been evident from their OD. All experiments were done in triplicate and the mean of three replicates were reported. 

### Statistical analysis

The student's t test was used for comparison of mean ofproliferation and viability between the contro (monolayer) and experimental (culture on scaffold) groups. The SPSS software version 16 (IBM SPSS Statistics, USA) was used for statistical analysis. Any P<0.05 was considered indicative of significant difference between groups. 

## Results

### Fabrication and characterization of fibrous scaffolds

**Fig.1:**
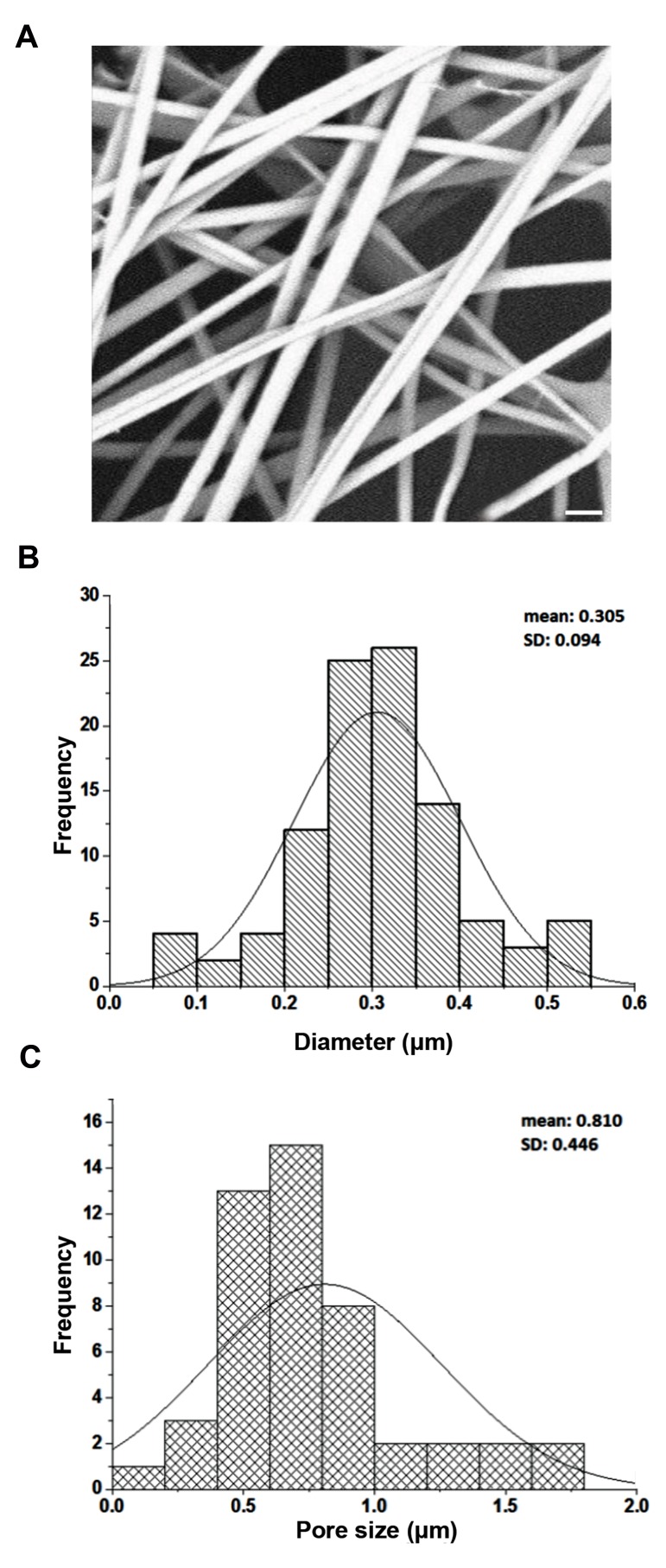
Physical analysis of electrospun scaffold. **A.** Scanning electron micrographof poly (vinyl alcohol)/human serum albumin/gelatin (PVA/HSA/gelatin) fibers(scale bar: 1 µm),** B.** Fiber diameter distribution of PVA/HSA/gelatin scaffold, and **C.** Fiber pore diameters of PVA/HSA/gelatin scaffold.

A PVA/HSA/gelatin homemade fibrous scaffold was designed by electrospinning for hTCs culture. Surface structure of composite fibers is shown in Figure 1A. Fiberdiameter was 100-600 nm (mean diameter 305 nm) (Fig .1B), and the average of pore sizes was 0.810 µm (Fig .1C). Surfacewettability as an important determinant of cell adhesion, proliferation, and migration, was also checked. The scaffolds were found to be hydrophilic with contact angles of 28.2° and 46.8°, before and after cross-linkage in glutaraldehyde vapor, respectively. Contact angledata supported the hypothesis that incorporation ofglutaraldehyde into scaffolds decreases hydrophilicity which consequently leads to higher biostability. 

### Fourier-transform infrared spectroscopy spectra

**Fig.2:**
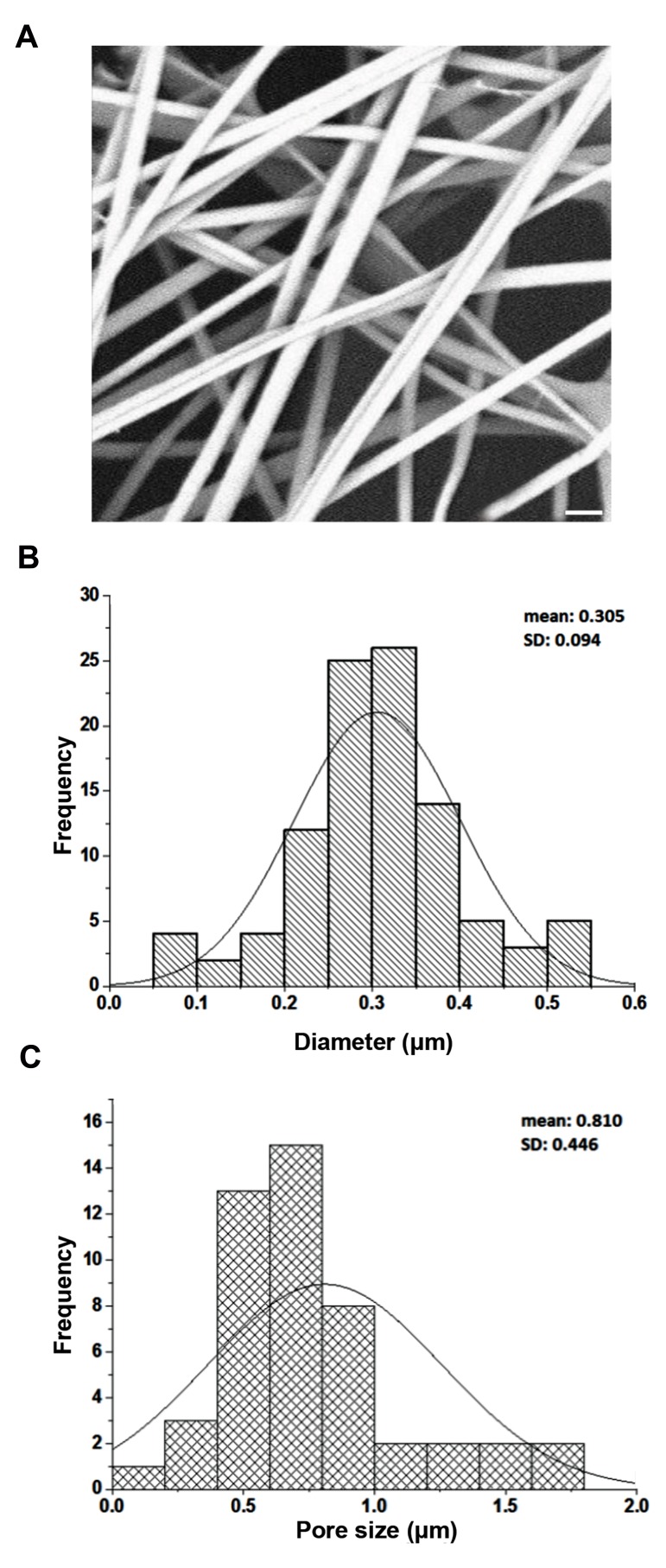
Fourier transform infrared spectroscopy of the scaffold and its constituents.

Chemical analysis of fibrous scaffold showed typical spectrum peaks for PVA, HSA and gelatin (Fig .2). The result for PVA showed absorption peaks at about 3200-3550 cm^-1^ (OH-OH stretching), 2930 cm^-1^ (C-H stretching), 1245 cm^-1^ (C-O stretching), 1084 cm^-1^ (C-O)-C-OH stretching), 937 cm^-1^ (CH-CH2stretching) and 865 cm^-1^ (C-C stretching). The result for gelatin showed absorption peaks at 1640-1636 cm^-1^ (amide I), 1544-1542 cm^-1^ (amide II), 1240 cm^-1^ (amide III) and 3300 cm^-1^ (amide A). The FTIR spectrum for HSA showed strong absorption peaks at 1550 cm^-1^ (amide I) and 1660 cm^-1^ (amide II). In the PVA/HSA/ gelatin fibers, very clear absorption peaks assigned to the PVA, were present at OH-OH and C-H stretching bands. Further typical absorptions were seen at amide I and amide II which can be assigned to the HSA as well as gelatin. These results indicated the presence of all three materials in the fiber. 

**Table 1:** Comparison of viability between testis cells cultured on scaffold and monolayer-cultured ones


Parameters	Optical density (7^th^ day)	Viability (7^th^ day)	Optical density (14^th^ day)	Viability (14^th^ day)
Group				

Monolayer	0.20 ± 0.1	-	0.23 ± 0.07	-
Electrospun scaffold	0.17 ± 0.06	85	0.18 ± 0.06	78.26


Data are presented as mean ± SD (for optical density, triplicate) and percentage (for viability).

### Isolation and culture of human testis-derived cells

During assessment of the spermatogenesis status of TESE samples, histological analysis of testicular tissue demonstrated the presence of somatic and germ cells in the tissue. Isolation of germ cells was not the aim of this study; we required just a few SSCs in the tissue as germ cells harboring stemness potential documented by H&E staining. 

The hTCs were initially floating, but began to attach after culture in the central dish. After one week, many of the cells were adherent and began to grow. After 5 passages, we had adequate numbers of cells to continue the study. 

### Morphology of human testis-derived cells

**Fig.3:**
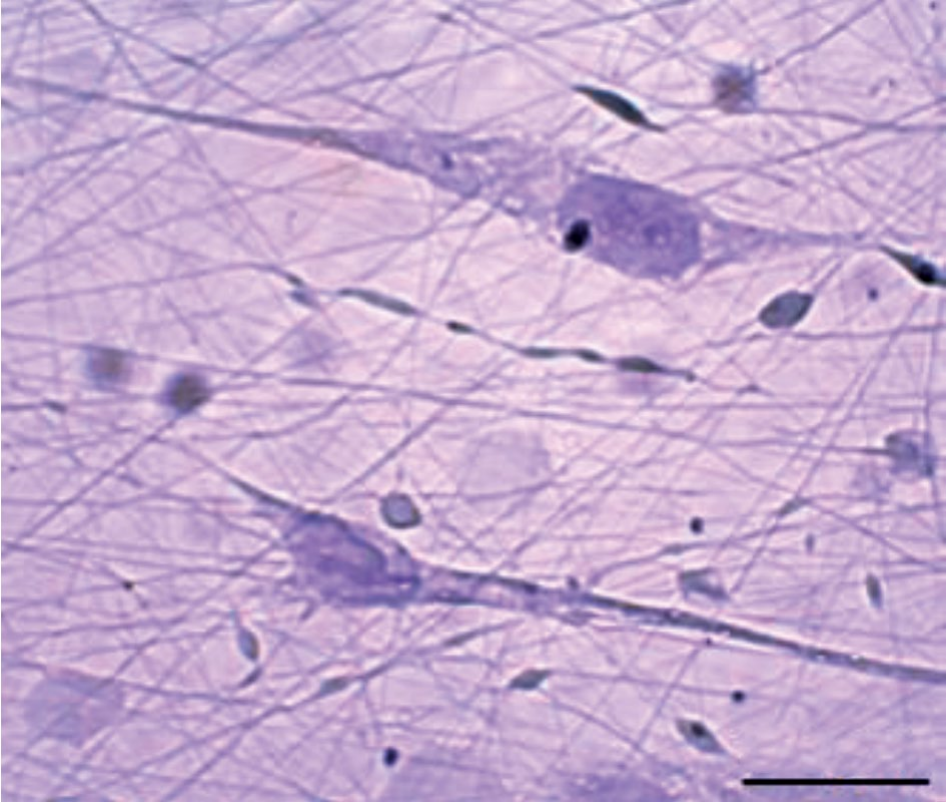
H&E staining of 7-day cultured cells seeded on poly (vinyl alcohol)/ human serum albumin/gelatin fibers (scale bar: 200 µm).

The presence of cells on scaffold was proved by SEM (not shown here). The results showed that the scaffold had the ability to support the hTCs during 14 days. Cells attachment to fibers and their normal shape were also demonstrated by H&E staining (Fig .3). It seems that this scaffold can mimic extracellular matrix (ECM).

### Proliferation and viability of human testis-derived cells

Table 1 shows the percentages of viable cells after 7 and 14 days of culture in both monolayer and scaffold-cultured groups treated with growth factors GDNF and EGF. About 85% of cells cultured on scaffold were viable on day 7 (in comparison with the monolayer control group which showed 100% viability), with a small drop in this figure at day 14 (78.26%). There was no significant difference (Student’s t test, P>0.05) in cell viability and proliferation rate between the control group and experimental groups based on MTT test results. Our data suggest the nontoxic nature of this scaffold for hTCs.

## Discussion

SSCs play crucial roles as male gamete (sperm) precursor cells which transfer father’s genetic information to the next generation. They are unipotent stem cells and their population in the testis is very small (20). Recently, in vitro production of haploid cells from SSC-like cells was shown in mice (21). Interestingly, in vitro production of functional sperms was confirmed by other studies using organ culture of SSC lines in neonatal mouse testis (22, 23). Nonetheless, there are ethical and practical challenges to achieve this aim in humans. Firstly, getting neonatal human testis biopsies to grow human SSCs is almost impossible. Secondly, despite the efforts made to isolate and expand human SSCs in culture to generate GSCs or human testis- derived embryonic-like stem cells (htESC-like cells) (13, 24, 25), some reports have indicated that these hTCs are not pluripotent and possess multipotent stromal characteristics (14).

It was shown in a study that induced pluripotent stem (iPS) cell-derived cells injected along with testicular cells into dorsal skin of mice are able to reconstitute seminiferous tubules, and iPS cell-derived germ cells can lodge at basement membranes of reconstituted tubules (26).

Tissue engineering methods using stem cells are applicable strategies in some problematic cases (27, 28). These methods can be used, for example, for synthesis of artificial ECM as a niche for cells in culture (29-31). In vivo, the SSCs are connected in some ways to other cells such as Sertoli and Leydig cells. This close proximity is very important for cells to exchange signals through secretion of growth factors (32). The scaffold should provide better conditions for the cells similar to those present in in vivo 3D condition (33). 

Previously, we showed construction of an artificial human testis using homemade human serum albumin and calcium phosphate 3D scaffolds coated with hTCs. Although histological structures similar to human seminiferous tubules were formed, but their arrangement was not comparable to that of the cells within the human testis (34).

Electrospinning is an applicable method used in drug delivery and tissue engineering. Different types of materials like poly (lactic acid)/chitosan, and PVA, have been used to make micro/nanofibers (18, 3539). In this study, human testicular cells were seeded on an electrospun PVA/HSA/gelatin fibrous mesh, to develop a 3D niche suitable for human male germ cells. HSA is the most abundant protein in human serum (35-50 g/L) with half-life of about 19 days. HSA was selected in this study because it is a very soluble globular monomeric protein besides being stable in the pH range of 4-9 and at high temperatures which is very critical in the process of making nanofibers. Temperature stability at near 60°C for up to 10 hours is necessary in this method. Another advantage is that when HSA is broken down, the resulting amino acids will nourish surrounding tissues. HSA is not only very cheap but also quite available. Finally, HSA has no toxicity and is biodegradable, two important points in regenerative medicine (40). Compared to the work on rat testicular cells seeded on poly(D,L-lacticco- glycolic acid) porous scaffolds which showed promising 75% viability up to 18 days and some degree of differentiation (39), our study on human testicular cells yielded 78% viability on the 14^th^ day.

In the present experiment, the initial number of cells cultured on each scaffold was 5000 cells. Since the supernatant of each microplate well was used for the MTT test, the optical densities reflect the number of cells. Since enumeration of the cells present on each scaffold was not easy, the only indicator of any proliferation or decrease in cell counts was the OD.

Since the OD of wells containing cells cultured on scaffolds were not significantly different from that of the monolayer cultures (used as the control group), we may conclude that they have proliferated only a little less than cells on the monolayer culture.

In our study, the viability and proliferation of the cells were examined by MTT assay and results indicated that this device is not toxic for the cells. SEM images showed homing of the cells within the fibers. Our data may serve as the starting point of human ambitions for recapitulation of human testis and probably other organs, with conceivable further applications in human developmental biology, toxicology, drug discovery and regenerative medicine. 

## Conclusion

In this study, a novel PVA/HSA/gelatin fibrous scaffold was designed and tested for physical, chemical and biologic properties, including its toxicity for hTCs. Promising performance of this scaffold in terms of biocompatibility and support of hTC growth encourages further evaluation of its in vivo ability to induce sperm production in animal models and then in human experiments.

## References

[1] Louis JF, Thoma ME, Sorensen DN, McLain AC, King RB, Sundaram R (2013). The prevalence of couple infertility in the United States from a male perspective: evidence from a nationally representative sample. Andrology.

[2] Evans MJ, Kaufman MH (1981). Establishment in culture of pluripotential cells from mouse embryos. Nature.

[3] De Felici M, McLaren A (1983). In vitro culture of mouse primordial germ cells. Exp Cell Res.

[4] Matsui Y, Zsebo K, Hogan BL (1992). Derivation of pluripotential embryonic stem cells from murine primordial germ cells in culture. Cell.

[5] Thomson JA, Itskovitz-Eldor J, Shapiro SS, Waknitz MA, Swiergiel JJ, Marshall VS (1998). Embryonic stem cell lines derived from human blastocysts. Science.

[6] Shamblott MJ, Axelman J, Wang S, Bugg EM, Littlefield JW, Donovan PJ (1998). Derivation of pluripotent stem cells from cultured human primordial germ cells. Proc Natl Acad Sci USA.

[7] Hübner K, Fuhrmann G, Christenson LK, Kehler J, Reinbold R, De La Fuente R (2003). Derivation of oocytes from mouse embryonic stem cells. Science.

[8] Clark AT, Bodnar MS, Fox M, Rodriquez RT, Abeyta MJ, Firpo MT (2004). Spontaneous differentiation of germ cells from human embryonic stem cells in vitro. Hum Mol Genet.

[9] Aflatoonian B, Moore H (2005). Human primordial germ cells and embryonic germ cells, and their use in cell therapy. Curr Opin Biotech.

[10] Aflatoonian B, Ruban L, Jones M, Aflatoonian R, Fazeli A, Moore HD (2009). In vitro post-meiotic germ cell development from human embryonic stem cells. Hum Reprod.

[11] Kanatsu-Shinohara M, Inoue K, Lee J, Yoshimoto M, Ogonuki N, Miki H (2004). Generation of pluripotent stem cells from neonatal mouse testis. Cell.

[12] Ryu BY, Orwig KE, Kubota H, Avarbock MR, Brinster RL (2004). Phenotypic and functional characteristics of spermatogonial stem cells in rats. Dev Biol.

[13] Mizrak SC, Chikhovskaya JV, Sadri-Ardekani H, van Daalen S, Korver CM, Hovingh SE (2010). Embryonic stem cell-like cells derived from adult human testis. Hum Reprod.

[14] Sadeghian‐Nodoushan F, Aflatoonian R, Borzouie Z, Akyash F, Fesahat F, Soleimani M (2016). Pluripotency and differentiation of cells from human testicular sperm extraction: An investigation of cell stemness. Mol Reprod Dev.

[15] Irie N, Surani MA (2017). Efficient induction and isolation of human primordial germ cell-like cells from competent human pluripotent stem cells. Method Mol Biol.

[16] Sato T, Katagiri K, Kubota Y, Ogawa T (2013). In vitro sperm production from mouse spermatogonial stem cell lines using an organ culture method. Nat Protoc.

[17] Ringe J, Kaps C, Burmester GR, Sittinger M (2002). Stem cells for regenerative medicine: advances in the engineering of tissues and organs. Naturwissenschaften.

[18] Won JJ, Nirmala R, Navamathavan R, Kim HY (2012). Electrospun core-shell nanofibers from homogeneous solution of poly (vinyl alcohol)/bovine serum albumin. Int J Biol Macromol.

[19] Li Y, Chen F, Nie J, Yang D (2012). Electrospun poly (lactic acid)/chitosan core-shell structure nanofibers from homogeneous solution. Carbohydr Polym.

[20] Dym M, Kokkinaki M, He Z (2009). Spermatogonial stem cells: mouse and human comparisons. Birth Defects Res C Embryo Today.

[21] Wang P, Suo LJ, Shang H, Li Y, Li GX, Li QW (2014). Differentiation of spermatogonial stem cell-like cells from murine testicular tissue into haploid male germ cells in vitro. Cytotechnology.

[22] Sato T, Katagiri K, Gohbara A, Inoue K, Ogonuki N, Ogura A (2011). In vitro production of functional sperm in cultured neonatal mouse testes. Nature.

[23] Sato T, Katagiri K, Kubota Y, Ogawa T (2013). In vitro sperm production from mouse spermatogonial stem cell lines using an organ culture method. Nat Protoc.

[24] Kossack N, Meneses J, Shefi S, Nguyen HN, Chavez S, Nicholas C (2009). Isolation and characterization of pluripotent human spermatogonial stem cell-derived cells. Stem Cells.

[25] Sadri-Ardekani H, Mizrak SC, van Daalen SK, Korver CM, Roepers-Gajadien HL, Koruji M (2009). Propagation of human spermatogonial stem cells in vitro. JAMA.

[26] Yang S, Bo J, Hu H, Guo X, Tian R, Sun C (2012). Derivation of male germ cells from induced pluripotent stem cells in vitro and in reconstituted seminiferous tubules. Cell Prolif.

[27] Caplan AI (2007). Adult mesenchymal stem cells for tissue engineering versus regenerative medicine. J Cell Physiol.

[28] Barrilleaux B, Phinney DG, Prockop DJ, O’connor KC (2006). Review: ex vivo engineering of living tissues with adult stem cells. Tissue Eng.

[29] Badylak SF (2002). The extracellular matrix as a scaffold for tissue reconstruction. Semin Cell Dev Biol.

[30] Li WJ, Laurencin CT, Caterson EJ, Tuan RS, Ko FK (2002). Electrospun nanofibrous structure: a novel scaffold for tissue engineering. J Biomed Mater Res.

[31] Lutolf MP, Hubbell JA (2005). Synthetic biomaterials as instructive extracellular microenvironments for morphogenesis in tissue engineering. Nat Biotechnol.

[32] Clermont Y (1972). Kinetics of spermatogenesis in mammals: seminiferous epithelium cycle and spermatogonial renewal. Physiol Rev.

[33] Saltzman WM, Parkhurst MR, Parsons-Wingerter P, Zhu WH (1992). Three-dimensional cell cultures mimic tissues. Ann NY Acad Sci.

[34] Borzouie Z, Hekmati-Moghadam SH, Talebi AR, Poor-Rajab F, Jebali A, Nikukar H (2015). Reconstitution of an artificial human testis using a 3 dimensional (3D) culture device.Proceedings of the 31st Annual Meeting of the European Society for Human Reproduction and Embryology, Lisbon. Hum Reprod.

[35] Sill TJ, von Recum HA (2008). Electrospinning: applications in drug delivery and tissue engineering. Biomaterials.

[36] Li Y, Chen F, Nie J, Yang D (2012). Electrospun poly (lactic acid)/chitosan core-shell structure nanofibers from homogeneous solution. Carbohydr Polym.

[37] Zhang C, Yuan X, Wu L, Han Y, Sheng J (2005). Study on morphology of electrospun poly (vinyl alcohol) mats. Eur Polym J.

[38] Eslahi N, Hadjighassem MR, Joghataei MT, Mirzapour T, Bakhtiyari M, Shakeri M (2013). The effects of poly L-lactic acid nanofiber scaffold on mouse spermatogonial stem cell culture. Int J Nanomedicine.

[39] Lee JH, Oh JH, Lee JH, Kim MR, Min CK (2011). Evaluation of in vitro spermatogenesis using poly (D, L‐lactic‐co‐glycolic acid)(PLGA)‐ based macroporous biodegradable scaffolds. J Tissue Eng Regen Med.

[40] Elzoghby AO, Samy WM, Elgindy NA (2012). Albumin-based nanoparticles as potential controlled release drug delivery systems. J Control Release.

